# Internal fixation of shaft humerus fractures by dynamic compression plate or interlocking intramedullary nail: a prospective, randomised study

**DOI:** 10.1007/s11751-014-0204-0

**Published:** 2014-11-19

**Authors:** Mir G. R. Wali, Asif N. Baba, Irfan A. Latoo, Nawaz A. Bhat, Omar Khurshid Baba, Sudesh Sharma

**Affiliations:** 1 Department of Orthopedics, Government Medical College, Srinagar, Srinagar, India; 2 Department of Orthopedics, Government Medical College, Jammu, Jammu, India

**Keywords:** Shaft humerus, Fracture, Intramedullary nailing, Plating

## Abstract

Compare the results of internal fixation of shaft of humerus fractures using dynamic compression plating (DCP) or antegrade interlocking intramedullary nail (IMN). Fifty patients with diaphyseal fracture of the shaft of the humerus and fulfilling the inclusion criterion were randomly assigned to one of the two groups. Twenty-five patients were managed with closed antegrade interlocking intramedullary nail, and 25 underwent open reduction and internal fixation using dynamic compression plating. The mean age of patients with IMN fixation was 37.28 years (SD 12.26) and 37.72 years (SD 12.70) for those who underwent plating. Road traffic accident was the most common mode of injury in both groups. There was a statistically significant difference between the two groups with respect to duration of hospital stay, operative time and blood loss. There was no significant difference between the two groups in terms of union or complications. The functional assessment at the end of 1 year between the two groups did not show any significant difference in outcome. Antegrade interlocking IMN and DCP fixation are comparable when managing diaphyseal shaft of humerus fractures with respect to union rates and complications. Although shoulder related complications are more in the IMN group, however, it is associated with shorter hospital stay, lesser operative time and less blood loss. This makes interlocking IMN an effective option in managing these fractures.

## Introduction

Fractures of the shaft of humerus are relatively common, representing 1–3 % of all fractures [1, 2]. Humerus shaft fractures are unique among all long bone fractures in having very good results with non-operative methods like hanging cast, functional brace, Velpeau dressing, coaptation splint and abduction cast [3–5]. Good functional outcomes in these fractures are partly due to the tolerance of malunion in humerus. However, all fractures are not amenable to conservative methods. The indications for operative treatment of the humeral shaft fractures include open fractures, segmental fractures, pathological fractures, fractures associated with vascular injuries, bilateral humerus fractures, polytrauma, radial nerve palsy after fracture manipulation, neurological loss after penetrating injuries, fractures with unacceptable alignment and failure of conservative treatment [2]. Non-operative treatment requires a long period of immobilization, which carries a risk of prolonged shoulder joint stiffness and inconvenience for the patient [6, 7]. Furthermore, non-union after conservative treatment of these fractures does occur in up to 10 % of the cases, and treatment of this condition can be very difficult [8–10].

There is a growing interest in treating even simple humeral shaft fractures by surgical modalities in order to avoid these problems and to allow earlier mobilization and rapid return to work [11, 12]. The usual operative methods involve the use of dynamic compression plate (DCP) or interlocking nail (ILN). Plate and screw fixation has traditionally been the preferred method and remains the gold standard for surgical management [13]. Use of the plate, however, requires extensive dissection and is complicated by the proximity of the radial nerve and the risk of mechanical failure in osteopenic bone. As a result of recent technical advances and success associated with nailing in other long bone fractures, there is a growing interest in the use of the humeral intramedullary nail for treating this fracture. ILN is a less invasive procedure with improved biomechanics and load-sharing feature of the implant. Fractures managed with ILN have better chances of union, as the surgery does not involve periosteal stripping and reaming produces act as an autograft. This benefit, however, comes at the cost of shoulder problems. We hypothesized that fractures managed by interlocking nailing would have faster union rates, less surgical time, shorter hospital stay, but more shoulder problems.

We compare the results of fixation of the humerus shaft fractures using either DCP or antegrade ILN with respect to hospital stay, blood loss, union time, functional results and complications associated with the procedure.

## Materials and methods

A prospective, randomized, comparative study of the management of acute humeral shaft fractures by antegrade interlocking nail fixation and dynamic compression plating was conducted in the Department of Orthopaedics, Government Medical College, Jammu. An informed consent was obtained from all the patients and approval of the hospital ethical committee was sought. Fifty consecutive shaft humerus fracture patients, presenting to the hospital and fulfilling the inclusion criterion were randomly assigned to either ILN group (Group A) or DCP group (Group B). All skeletally mature patients with displaced fractures of humeral shaft, <3-week-old trauma, and requiring surgery were included. Skeletally immature patients, pathological fractures, compound fractures, associated neurovascular injuries, segmental fractures, radial nerve injury following closed reduction, non-cooperative patients and patients with other pathologies of the upper extremities were excluded. However, patients with pre-operative radial nerve injury were included in the study (except those where radial nerve palsy developed following manipulation). None of the patients necessitated intra-operative change in the procedure.

After complete pre-operative assessment and anaesthetic clearance, patients were randomized to receive either dynamic compression plating or interlocking nail, for definitive fracture fixation. AO classification system was used to classify the fractures. All surgeries were performed by surgeons (SS and MGR), familiar with both the procedures. In the plating group (group B), fixation was done with 4.5-mm dynamic compression plates using appropriate surgical techniques, depending on the fracture configuration. Transverse or short oblique fractures were stabilized by axial compression, while in the spiral or oblique fractures interfragmentary lag screw fixation was done, followed by application of plate in the neutralization mode. Anterolateral or posterior approach was used, depending upon the fracture configuration and the surgeon preference. Fixation of at least six cortices, preferably eight cortices, both proximal and distal to the fracture was obtained in every patient.

In group A (ILN), commercially available reamed antegrade interlocking nails (Nebula Surgicals, Gujarat, India) were used. The nails had a 5° bend in the proximal part. The nail was used because of the easy availability and economy. The nail had two screws proximally and two distally. One proximal screw was oriented transversely and the other obliquely, while one distal screw was directed anteroposteriorly and the other transversely. A 4–5 cm incision, lateral to the acromion, was made to facilitate the splitting of the deltoid muscle. The posterior margin of the greater tuberosity was exposed by retracting the supraspinatus tendon. The entry hole was made with an awl. The canal was gradually enlarged by reaming after insertion of a guide pin. During reaming, cortical contact at fracture site was ensured to prevent radial nerve injury. After passing the nail in the canal, fracture site was inspected under image intensifier to avoid distraction at the fracture site. The distal screws were fixed by the freehand technique. To prevent damage to the neurovascular structures, the entry holes were visualized by image intensifier followed by stab incision and blunt dissection to the bone. The proximal screws were fixed by the target device.

The blood loss was calculated from a modification of the Gross formula given below [14]:

$$\text{Blood loss}=\text{Blood volume}\left[\text{Hct}\left(\text{i}\right)-\text{Hct}\left(\text{f}\right)\right]/\text{Hct}\left(\text{m}\right)$$

where blood volume = body weight (kgs) × 70 ml/kg; Hct (i), Hct (f) and Hct (m) were the initial, final and mean (for final and initial) haematocrits, respectively.

Post-operative radiographs were checked to know the adequacy of reduction and any iatrogenic complication. Post-operatively the limb was placed in an arm sling and pendulum and elbow movements were allowed on the second post-operative day. Patients were discharged once they became comfortable, wound was healthy and the patient was afebrile. Patients were followed up at 2, 6, 12, 24, 36 weeks and final follow-up at 52 weeks. On each follow-up, the patients were examined clinically to check for signs of infection, pain, range of motion of elbow and shoulder, neurovascular status and any other complication. Radiological assessment using plain radiographs was done to know the status of union of the fracture, alignment, hardware problems and any malunion. The functional results at the end of 1 year were assessed using the American Shoulder and Elbow Surgeons (ASES) score for 13 activities of daily living requiring the shoulder and elbow movement with each activity carrying a maximum of 4 points. Radiological union was defined as the presence of bridging callus in two planes (AP and lateral). Union was defined as fracture healing within 4 months, delayed union as no signs of union 4–6 months of injury and non-union as no signs of union after 6 months. Rodríguez–Merchán criteria were used to assess the final results [15]. This criterion includes the assessment of shoulder and elbow range of movement, pain and disability. When the criteria fall in different categories, the lower category is used to classify the outcome (Table 1). The results were analysed statistically using the SPSS 16.0 software with the Student’s *t* test. The value of alpha was set to 0.05. The sample size was calculated after a literature review of previous similar studies.

**Table 1:** Criteria for evaluating functional results (Rodríguez–Merchán)

Rating	Elbow range of movement	Shoulder range of movement	Pain	Disability
Excellent	Extension 5° flexion 130°	Full range of movement	None	None
Good	Extension 15° flexion 120°	<10 % loss of total range of movement	Occasional	Minimum
Fair	Extension 30° flexion 110°	10–30 % loss of total range of movement	With activity	Moderate
Poor	Extension 40° flexion 90°	>30 % loss of total range of movement	Variable	Severe

## Results

In our study, 25 patients of fracture shaft of humerus were treated with antegrade ILN and 25 more cases underwent DCP fixation. The ILN group comprised 21 male and 4 female patients with mean age of 37.28 years (SD 12.26), while the DCP group had 20 males and 5 females having mean age of 37.72 years (SD 12.70) (*p* value > 0.05). In the ILN group, 16 patients (64 %) had AO type A fracture, 6 (24 %) had AO type B and 3 (12 %) patients had AO type C fracture. The pattern was similar in DCP group with 17 patients (68 %) having type A, 6 (24 %) patients type B and 2 (8 %) patients had type C fracture. In our study, both the groups were comparable with respect to the level of fracture. Majority of the fractures in both the groups were in the middle third of the shaft of humerus. However, the next commonly involved level in DCP group was distal third (24 %) of shaft, compared with ILN group, where next commonly involved level was proximal third (24 %) of shaft.

Road traffic accident (RTA) was the most common mode of injury in majority of patients in both the groups, followed by fall from height and direct trauma to arm. In the DCP group, 18 patients (72 %) had RTA, 5 (20 %) had fall from height and two patients sustained direct trauma to arm. In the nailing group, 19 patients had RTA, 4 had fall from height and two sustained direct trauma to arm. The most common associated injuries were other long bone fractures, followed by head injury, pelvic trauma and chest injury (Table 2). The mean interval between admissions to surgery was 6.12 days (SD 3.67) in the ILN group and 11.88 days (SD 3.29) in the DCP group, the difference between the two being statistically significant (*p* value < 0.05). In the DCP group, 17 patients were operated using the anterolateral approach and remaining 8 patients with the posterior approach. The average operating time in the ILN group was 50.8 min (SD 6.87) and 66.2 min (SD 8.07) in the DCP group, the difference again being statistically significant (*p* value < 0.05). The average blood loss in the ILN group was 140 ml (range 90–550), while the average loss in DCP group was 310 ml (range 160–880), the difference being statistically significant. The duration of hospital stay was 8.76 days in ILN group and 14.56 days in the DCP group, the finding again being statistically significant. The mean fluoroscopy time in the interlocking group was 4.6 min, while fluoroscopy was not used in the plating group.

**Table 2:** Associated injuries

	Other long bone fractures	Head injury	Chest trauma	Pelvis injury	Abdominal injury
DCP group	2	2	1	1	0
ILN group	4	1	2	1	1

**Fig. 1:**
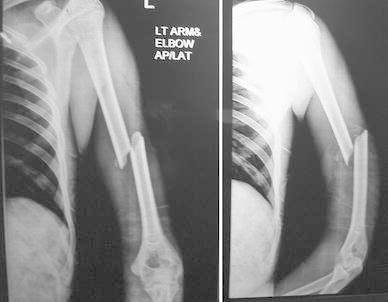
AP and lateral radiographs of a fracture shaft of humerus in the middle third

**Fig. 2:**
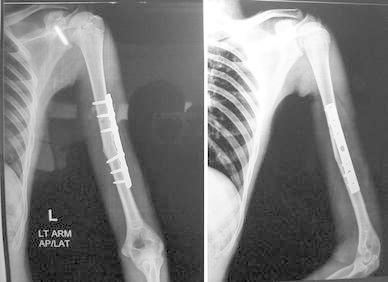
AP and lateral radiographs of the fracture in Fig. 1 shows solid union with DCP after 9 months

**Fig. 3:**
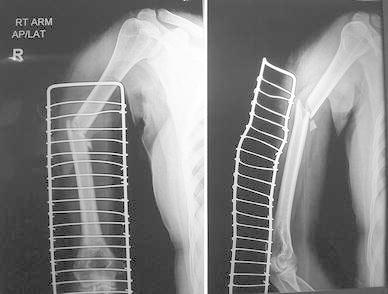
AP and lateral radiographs of fracture middle third of the shaft of humerus

**Fig. 4:**
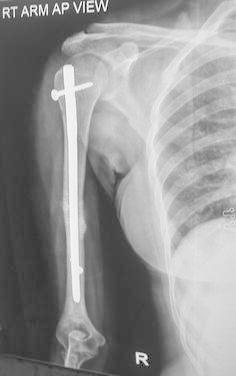
AP view of fracture shows good union with IMN

**Fig. 5:**
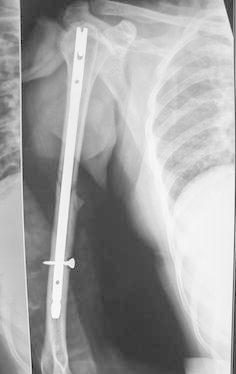
Lateral view of fracture shaft of humerus shoes uniting fracture with the orientation of distal screw

Most of the fractures united within 16 weeks in both the groups (Figs. 1, 2, 3, 4, 5). Union was defined as the presence of bridging callus in two planes and the absence of pain and movement at fracture site. Three patients in nailing group and two in plating group had delayed union and united between 4 and 6 months. Two patients in ILN group (8 %) and two patients (8 %) in the DCP group did not show signs of union till 6 months (Table 3). One patient in nailing group had iatrogenic comminution at the fracture site with distraction at the fracture site. Both patients in the nailing group underwent nail removal and plating with bone grafting, and the two patients in DCP group underwent bone grafting as a secondary procedure. All the fractures went on to eventual union. The mean duration of union in remaining patients in ILN group was 13.60 (SD 4.32) weeks and in DCP group was 15.2 (SD 5.65) weeks. Although average union time in ILN group was 1.6 weeks earlier than DCP group, the finding was not statistically significant (*p* value 0.376).

**Table 3:** Time to union (weeks)

	Up to 8	8–12	13–16	17–24	>24
IMN (*n* = 25)	5	9	6	3	2
Plating (*n* = 25)	2	10	9	2	2

Pre-operative radial nerve palsy was seen in two patients in ILN group and one patient in DCP group. The radial nerve was explored only in the DCP group and found intact. All the three patients completely recovered. Two patients in plating group developed post-operative radial nerve injury. One of the patients agreed for exploration, and the radial nerve was found stuck beneath the plate. One patient in the ILN group developed iatrogenic comminution at the site of nail entry, but this did not affect the final outcome. No patient in the plating had hardware failure in the form of plate bending or screw backout.

**Fig. 6:**
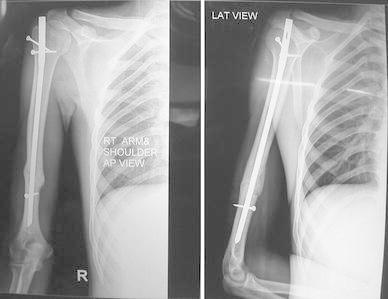
AP and lateral view of a united fracture shows proximal protrusion of the nail

In the ILN group, shoulder stiffness was the most common complication occurring in 4 patients (16 %). Of these, stiffness resolved with physiotherapy in three patients and one patient continued to have stiffness. One patient in ILN group had severe shoulder impingement due to the protruding nail which required removal of the nail after achieving union (Fig. 6). This was one of the earliest cases in the series, and despite using C-arm, we were not able to appreciate the protrusion. One patient each, in both the groups, had elbow stiffness, while one patient in the plating group developed shoulder stiffness. One patient in nailing group developed superficial infection at the nail entry site which resolved with antibiotics. Two patients (8 %) in the plating group developed superficial infection which was resolved with antibiotics, and one patient developed deep infection which required serial debridement and antibiotics. Three patients in the nailing group and four in the plating group required repetition of operation (Table 4).

**Table 4:** Post-operative complications

	Superficial infection	Deep infection	Radial nerve palsy	Comminution at fracture site	Shoulder stiffness	Elbow stiffness	Delayed union	Non-union
IMN	1	0	0	0	4	1	3	2
DCP	2	1	2	1	1	1	2	2

The functional assessment after 1 year of surgery using the ASES score did not reveal any significant difference between the two groups. ASES score in the ILN group was 43.2 and in the DCP group was 44.1. The final evaluation of ILN patients done with the Rodríguez–Merchán criteria revealed excellent results in 7 (28 %), good in 13 (52 %), fair in 3 (12 %) and poor in 2 patients. Results were similar in the DCP with excellent result in 8 (32 %), good in 13 (52 %), fair in 2 and poor in 2 patients.

## Discussion

Humerus fracture is unique amongst the long bone fractures in its tolerance of less than anatomical reduction. Shortening up to 3 cm, rotation <30° and angulation up to 20° are considered acceptable [16]. Due to this fact, most of the humerus fractures are still managed conservatively and have good functional results. The most common indication of operative intervention is inability to achieve acceptable reduction, followed by associated vascular lesions, open fractures, radial nerve palsy, polytrauma patients, floating elbow and pathological fractures [17]. The preponderance of the fracture in young males, commonly in third and fourth decade of life, was seen in our series, as has been reported by other similar studies [18]. Road traffic accident is the most common mode of injury, especially in younger patients.

In the past, open reduction and plating was the preferred method of operative intervention, and still continues to remain the gold standard. However, conventional plating technique involves an extensive surgical approach for open reduction of fracture and is theoretically associated with increased risk of radial nerve injury and more blood loss. The excellent result of intramedullary interlocking nails in tibia and femur fractures has stimulated interest in applying the same methods in humerus fractures. Intramedullary nails are subjected to smaller bending loads than plates because they are closer to the mechanical axis than the usual plate position on the external surface of the bone. Intramedullary nails can also act as load-sharing devices in fractures with cortical contact. Moreover, the stress shielding commonly seen with plates and screws is minimized with intramedullary nails. Intramedullary nailing in humerus fractures is a less invasive procedure which maintains the biology and gives a good, stable fixation. It is also assumed to result in quicker union, less blood loss and less chances of radial nerve injury. However, controversy still exists over the best method of fixation. Ooyung et al. [19] in a meta-analysis of ten studies comparing the results of plating versus nailing concluded that both achieve similar results in humerus fractures, but plating was associated with reduced shoulder problems.

Union rates in our study were comparable between the two groups, non-union being seen in 8 % in ILN and 8 % in DCP group. Similar rates of non-union have been observed in most of the studies [20, 21]. Non-union in the DCP group is usually due to extensive soft tissue dissection or malreduction and is often associated with implant failure. Even though the effect of reaming might facilitate bone healing, non-union has been reported in 0–9 % of cases [22, 23] managed with reamed intramedullary nails. Non-union in ILN usually results from distraction at the fracture site as humerus seems to be less forgiving than tibia or femur in this aspect. Non-union in both groups was managed by open reduction and plating with bone graft, as has been suggested in the literature [24]. The time to achieve union in our study was less in ILN than in plating group, although not statistically significant. Denies et al. [25] also reported earlier union in nailing, probably due to the less invasive nature of the procedure and the maintenance of the fracture haematoma. Changulani et al. [26] also reported earlier union in nailing with a statistically significant difference.

Whether to use reamed or unreamed nails is still a controversial topic. The advantages of reaming include the significant increase in the blood flow to the muscles and surrounding soft tissues, and this increase persists for up to 6 weeks. The increase in soft tissue blood flow may increase the cortical blood flow and thus more chances of union. Further, the cost of reamed nails is less, which is an advantage for developing nations, like ours. The disadvantage of reaming is the chances of radial nerve injury, especially when there is a gap at the fracture site. Further, some studies have shown extensive heat necrosis from nailing in small diameter canals. Reaming in small diameter canals may lead to distraction at the fracture site.

Infection, iatrogenic radial nerve palsy and hardware failure are most important complications associated with plating. We had higher rates of infection and iatrogenic radial nerve palsy in the plating group, as has been seen by Changulani et al. [26]. However, in a meta-analysis, Bhandari et al. [27] did not show higher risks of infection or radial nerve palsy with plating. Radial nerve is at a definite risk in plating, with special precautions being taken to prevent nerve from coming beneath the plate. Although radial nerve injury after nailing is rare, the risk can be further minimized by ensuring accurate reduction of the fracture (no gap) before passage of the reamers or the nail and by avoiding reaming in areas of comminution where the nerve is closely apposed to the bone.

Impairment of shoulder function is the main drawback of interlocking nailing. Shoulder pain in these patients may be related to violation of the rotator cuff, prominent nail end, adhesive capsulitis or unknown causes [27, 28]. We had shoulder problems in 20 % of our patients. One patient with protruding nail required a second surgery for the removal of implant. Chao et al. [28] also reported three patients with proximal protrusion of the nail. This usually arises from not pushing the nail distal enough, possibly from fear of producing a distal fracture, or from migration of an unlocked nail. We suggest assessing the length accurately before passing the nail and using C-arm till the procedure ends. Similar findings have been reported by many studies [28, 30, 31]. However, Flinkilla in an analysis of shoulder from different studies reported similar shoulder scores in both nailing and plating groups, with plating having better abduction and flexion [32]. We had intra-operative comminution at fracture site in one patient where the medullary canal was narrow which resulted in distraction at fracture site and eventually resulting in non-union. Introduction of large reamers or nail into a narrow canal can result in comminution at fracture site which are usually undisplaced fissures not requiring fixation. The incidence of this complication has decreased from 6 to 1.8 % due to introduction of newer nail designs [33]. Re-operation rates were similar between the two groups as has been seen by Denies et al. [25]. However, Bhandari et al. [27] reported more re-operations in the nailing group. Indications of re-operation in plating are union problems, hardware failure and revision for radial nerve palsy while the nailing patients it is for union problems, removal of protruding implant and management of preoperative fracture. A disadvantage of nailing is the need of fluoroscopy for the procedure and the associated risks to the surgeon and the theatre personnel. The fluoroscopy time reported by us is comparable to that reported in the literature and seen for other long bone fractures. To overcome this, expandable nails have come into the market which require less extensive use of fluoroscopy.

In our study, nailing was superior to plating with respect to the average post-operative stay of the patients and operating time. The main reason for the longer stay in the plating group in our study was because of the longer delay in surgery in the plating patients. This was due to our tendency to do DCP only when the swelling had completely subsided. In contrast, ILN does not need rigorous subsidence of the swelling. Most of our patients belonged to far-off places where sterile-dressing facilities were not readily available and thus tended to stay till the operative wound was deemed clean. ILN patients had an edge due to their smaller surgical wounds and so were discharged earlier. The shorter stay, with a less invasive method, such as closed nailing, is of great advantage in developing countries where the orthopaedic hospital beds are limited and resources are scarce. For the same reason, the less operating time reported by us is also advantageous. Chao et al. also had shorter operative time in ILN, although the difference was not significant, while Chaudhary et al. had shorter operative time in the plating group [29, 34]. ILN was also associated with significantly decreased blood loss than plating, as has been seen in most of the studies [29]. Although this difference is statistically significant, but in the clinical settings, this difference is marginal.

The ASES score and the final outcome in our series did not show any significant advantage of one method over the other. Some studies have shown plating to be more effective, while others have found better results with nailing [35, 36]. However, the meta-analysis of different randomized and quasi-randomised controlled trials comparing the two failed to find any significant difference between the two with respect to ASES scores [37].

The most important factors in obtaining fracture healing are anatomical reduction, stable fixation and adequate blood supply. Although internal fixation with DCP may result in a better reduction, it also carries a more extensive soft tissue dissection with risk of radial nerve lesion and infection. ILN provides secure and rigid fixation with less soft tissue damage and maintaining the biology. Although ILN is associated with relatively increased incidence of shoulder complications, it has definite advantages in terms of shorter hospital stay, less blood loss and shorter operative time, which are of immense importance in the developing countries with limited resources.

We conclude that antegrade locked intramedullary nailing is an effective alternative to plating in shaft humerus fractures as it has comparable results in terms of union rate and complications. In addition, it has the added advantage of lesser operative time and shorter hospital stay, both of which have a distinct advantage in those centres in developing countries which have the facility of fluoroscopy.
